# Age- and body surface area-specific ultrasound percentile charts for thyroid volume in Polish school-aged children: comparison with European cohorts

**DOI:** 10.3389/fendo.2026.1945848

**Published:** 2026-09-07

**Authors:** Andrzej Nowak, Małgorzata Trofimiuk-Müldner, Jacek Podlewski, Monika Buziak-Bereza, Grzegorz Sokołowski, Marek Ruchała, Zbigniew Adamczewski, Arkadiusz Zygmunt, Krzysztof Sworczak, Alicja Hubalewska-Dydejczyk

**Affiliations:** 1 Department of Endocrinology, Endocrine Oncology, Nuclear Medicine and Internal Diseases, University Hospital in Krakow, Kraków, Poland; 2 Jagiellonian University Medical College, Chair and Department of Endocrinology, Kraków, Poland; 3 Dover Fueling Solutions, Kraków, Poland; 4 Department of Endocrinology, Metabolism and Internal Medicine, Poznan University of Medical Sciences, Poznań, Poland; 5 Department of Nuclear Medicine, Medical University of Lodz, Łódź, Poland; 6 Department of Endocrinology and Metabolic Diseases, Polish Mother’s Memorial Hospital-Research Institute, Łódź, Poland; 7 Department of Pediatric and Adult Endocrinology, Medical University of Lodz, Łódź, Poland; 8 Department of Endocrinology and Internal Medicine, Medical University of Gdansk, Gdańsk, Poland

**Keywords:** body surface area, children, iodine sufficiency, percentile charts, Poland, thyroid volume, ultrasound, urinary iodine concentration

## Abstract

**Objective:**

To establish age- and body surface area (BSA)-specific ultrasound percentile charts for thyroid volume (Tvol) in Polish school-aged children born after the introduction of mandatory iodine prophylaxis, and to compare these values with previous national, WHO, and recent European reference data.

**Design:**

Nationwide cross-sectional analysis of iodine-monitoring data. Methods: The source database contained 10,456 screening records from the National Program for the Elimination of Iodine Deficiency in Poland. After hierarchical exclusions, 3,170 children aged 6–13 years with valid thyroid-volume data were included in the age-specific analysis; 2,843 children with complete anthropometric data were included in analyses requiring BSA. Age- and BSA-specific percentile curves (P3-P97) were generated, and multivariable linear regression was performed to identify independent predictors of Tvol.

**Results:**

Median urinary iodine concentration (UIC) exceeded 100 µg/L across all age groups, confirming iodine sufficiency at the population level. Median Tvol increased with age from 2.7 to 7.4 mL in girls and from 2.8 to 6.8 mL in boys. The 97th percentile increased from 4.5 to 12.9 mL in girls and from 4.3 to 13.6 mL in boys. BSA was the strongest independent predictor of Tvol, followed by age. Compared with WHO 2004 reference values, the proposed percentiles were consistently higher and closely aligned with recent European data.

**Conclusions:**

This study provides population-specific ultrasound percentile charts for thyroid volume in iodine-sufficient Polish children. These charts complement existing WHO and European references and may improve the interpretation of pediatric thyroid ultrasound in Poland and comparable Central European populations.

## Introduction

The assessment of thyroid volume (Tvol) serves as a crucial metric in monitoring iodine nutrition in populations. In children, thyroid enlargement may reflect both physiological growth and the harmful impact of environmental or nutritional factors, including iodine insufficiency (1, 2). While urinary iodine concentration (UIC) reflects current iodine intake, Tvol indicates the gland’s adaptive response to long-term iodine exposure and is therefore a valuable complementary indicator of iodine sufficiency (3, 4). Because thyroid palpation has low sensitivity and specificity, ultrasound has become the preferred diagnostic tool for assessing thyroid morphology and volume. Its non-invasive nature and reproducibility make it ideal for pediatric populations, provided that valid reference values are available (5–7).

Although the current World Health Organization (WHO) reference values for Tvol in children, established in 2004, are still widely used as the international standard, several studies have recommended developing population-specific, regionally derived reference charts to account for differences in ethnicity, anthropometry, and iodine-supply history (3, 5).

Currently used Polish pediatric reference values for Tvol were established more than a decade ago and were derived from a relatively small regional sample (8). In addition, previous Polish charts did not provide BSA-specific percentile estimates. Since then, secular changes in growth and body composition have been observed among Polish schoolchildren, including increases in body surface area (BSA) compared with earlier generations (9).

The aim of this study was to derive age- and BSA-specific ultrasound reference values for Tvol from a large cohort of Polish school-aged children born after the implementation of mandatory table salt iodization, and to compare these values with previous Polish, WHO, and recent European reference data.

## Materials and methods

The data were collected between 1999 and 2017 as part of the National Program for the Elimination of Iodine Deficiency in Poland. The source database contained 10,456 screening records from Polish schoolchildren examined across Poland. After exclusion of seven records with missing or invalid total thyroid volume, 10,449 records with valid Tvol were available for cohort construction.

The analytical cohort was constructed hierarchically. We retained children born after December 31, 1997, aged 6–13 years at examination, with BSA ≤1.8 m^2^ for BSA-specific analyses, without focal lesions on thyroid ultrasonography (e.g., thyroid nodules or cysts), and with negative history of thyroid disease, thyroid surgery, levothyroxine use, or anti-thyroid drug use.

Only children with written informed consent from their parents or legal guardians were included.

Eligibility was further restricted to participants from iodine-sufficient regions, defined by median urinary iodine concentration (UIC) ≥100 µg/L in the corresponding school or region. The full exclusion cascade is shown in **Table 1**.

**Table 1:** Participant flow-table and construction of analytical cohorts.

Step	Criterion/reason for exclusion	N before	Excluded n	Remaining n	% retained from previous step	% of source database retained
0	Source screening records	–	–	10,456	–	100.0%
1	Missing or invalid total thyroid volume	10,456	7	10,449	99.9%	99.9%
2	Date of birth before 1998	10,449	5,615	4,834	46.3%	46.2%
3	Age at measurement below 6 or over 13 years	4,834	19	4,815	99.6%	46.1%
4	BSA >1.8 m^2^	4,815	64	4,751	98.7%	45.4%
5	Thyroid cysts	4,751	44	4,707	99.1%	45.0%
6	Thyroid nodules	4,707	41	4,666	99.1%	44.6%
7	Levothyroxine or anti-thyroid drug use	4,666	107	4,559	97.7%	43.6%
8	Region with median UIC <100 µg/L	4,559	1,389	3,170	69.5%	30.3%
9	Missing BSA (only for analyses requiring BSA)	3,170	327	2,843	89.7%	27.2%

Percent retained from the previous step was calculated as N after/N before × 100. Percent of source database retained was calculated relative to the 10,456 source screening records. The final age-specific Tvol cohort included 3,170 children; analyses requiring BSA used the BSA-complete subset of 2,843 children. UIC, urinary iodine concentration; BSA, body surface area; Tvol, thyroid volume.

A total of 3,170 children who met these criteria and had complete data on thyroid volume were included in the age-specific Tvol analysis (**Table 1**). A slightly smaller sample of 2,843 children was included in analyses requiring complete BSA data.

Sex was recorded as female or male in the original database. The study population was ethnically homogeneous, with all participants (100%) being of Caucasian origin. The study was approved by the local Ethics Board (1072.61201.7.2017).

### Ultrasound, anthropometric and laboratory assessment

Basic anthropometric measurements were performed in 2,843 children. Body weight and height were assessed in light clothing according to standardized procedures. BSA was calculated using the Du Bois formula: BSA (m^2^) = weight (kg)^0.425 × height (cm)^0.725 × 0.007184 (10). Tvol was determined with a Siemens Sonoline Prima ultrasound device equipped with a 7.5 MHz, 6-cm linear transducer mounted in the mobile “Thyromobil” unit (Merck KGaA, Darmstadt, Germany). Examinations were carried out in the supine position with the neck extended. The width and depth of each lobe were obtained from transverse sections, and the length from longitudinal sections. The isthmus and capsule were excluded. Lobe volume was calculated as width × depth × length × 0.479 (mL), according to the Brunn formula, and total Tvol was expressed as the sum of both lobes (11).

UIC was measured in casual morning urine samples using the Sandell–Kolthoff colorimetric method. Urine samples were stored at –20 °C until analysis.

### Statistical analysis

Analyses were performed in R version 4.4.2. The GAMLSS package v.5.4.22 (12) was used to calculate centile curves. The emmeans package v.1.10.6 (13) was used to test adjusted sex differences, and ppcor v.1.1 (14) was used to calculate semi-partial correlations. In all statistical tests, a significance level of 0.05 was applied. Confidence intervals for the median were calculated using bootstrap resampling. Confidence intervals for multiple age-group comparisons were adjusted using Holm’s method.

We performed winsorization of Tvol. Outliers were identified as values exceeding median ± 2.5 times the median absolute deviation (MAD) and were replaced by median ± 2.5 times the MAD. The data were separately winsorized according to age and BSA, with median and MAD calculated separately for different age or BSA groups. Age-stratified winsorization affected 85 observations (2.7%), whereas BSA-stratified winsorization affected 98 observations (3.4%).

Age- and sex-specific percentile reference values for Tvol were derived using the lambda-mu-sigma (LMS) method implemented in the GAMLSS R package. This method models the dependent variable as a statistical distribution with three parameters: L (λ, for skewness/shape), M (μ, location parameter), and S (σ, scale parameter), which are smooth functions of the predictor variable. The reference values are then obtained as quantiles of this distribution, with parameters calculated for selected input values.

We modeled Tvol as a Box-Cox Cole and Green distribution (15). We compared different smoothing techniques available and finally chose monotonic P-splines to model the dependence of LMS parameters on BSA, and cubic splines to model the dependence on age, as these techniques demonstrated the best fit to the data, measured with generalized Akaike criterion (GAIC) and proper smoothness of resulting curves, verified visually. The degree of smoothing was chosen automatically using a built-in procedure from the GAMLSS package. All analyses were separately performed for age and BSA by sex. For all LMS models, the fit was assessed using extensive diagnostic tools available in the GAMLSS package, such as Q-Q plots and worm plots.

The normative values for Tvol obtained in our Polish cohort were compared with previously published Polish data, the current WHO reference charts (1997/2004), and other contemporary European studies.

Multiple linear regression was performed to identify independent predictors of Tvol. The final model included BSA, sex, and age as covariates. Individual spot UIC was summarized descriptively but was not retained in the final regression model, because iodine sufficiency was defined at the school or regional level using median UIC. An additional model replacing BSA with BMI was estimated to assess whether BMI improved anthropometric scaling. Semi-partial correlations included in the regression summary were calculated using the ppcor R package v.1.1 (14). The complete BMI-based regression output is provided in **Supplementary Table 2**.

We additionally developed a multivariable predictive model for Tvol distribution based on an extended LMS framework, enabling percentile estimation according to age, sex, and anthropometric parameters. This approach may facilitate individualized clinical assessment of thyroid volume. A prototype tool was implemented using R Shiny (**Supplementary Figure 1**).

## Results

A total of 3,170 children (1,604 girls and 1,566 boys aged 6–13 years) born after 1997 were included in the age-specific Tvol analysis (**Table 1**). This cohort represented 30.3% of the 10,456 source records and 30.3% of records with valid Tvol. Age- and sex-specific Tvol percentiles (P3, P10, P25, P50, P75, P90, P97) were calculated. Anthropometric characteristics are shown in **Table 2** for the BSA-complete subset (n=2,843), which represented 89.7% of the final Tvol cohort. Additional characteristics of the final analytical cohort and selected excluded subgroups are provided in **Supplementary Table 1**.

**Table 2:** Anthropometric characteristics of Polish school-aged children by age and sex (girls and boys).

Girls (n=1420)							
Age	n	BW [kg]		H [cm]		BSA [m^2^]	
		X̅ ± SD	Median (95% CI)	X̅ ± SD	Median (95% CI)	X̅ ± SD	Median (95% CI)
6	55	23.28 ± 4.25	22.6 (21.4 - 24)	120.35 ± 4.81	120 (118 - 120)	0.88 ± 0.08	0.88 (0.85 - 0.89)
7	263	25.36 ± 4.72	24.5 (23.5 - 25.7)	125.25 ± 5.64	125 (123 - 125)	0.94 ± 0.1	0.92 (0.91 - 0.94)
8	295	28.74 ± 6.25	28 (27 - 28)	130.54 ± 5.77	130 (129 - 129)	1.02 ± 0.12	1.01 (0.99 - 1.01)
9	224	32.79 ± 7.34	31.25 (30.05 - 32.6)	136.67 ± 6.65	136 (134 - 137)	1.11 ± 0.14	1.1 (1.08 - 1.12)
10	200	38.37 ± 9.51	35.4 (34 - 38)	143.06 ± 6.81	143 (141.5 - 144)	1.23 ± 0.16	1.21 (1.18 - 1.23)
11	166	40.12 ± 8.77	38.5 (37 - 39.5)	147.97 ± 7.21	148 (145.5 - 148)	1.29 ± 0.15	1.27 (1.24 - 1.3)
12	174	46.26 ± 9.69	44 (42 - 44)	154.45 ± 6.87	155 (153 - 156)	1.41 ± 0.16	1.39 (1.37 - 1.42)
13	43	46.02 ± 8.59	45 (39 - 45)	158.12 ± 5.26	159 (157 - 160)	1.43 ± 0.13	1.44 (1.38 - 1.45)

**Table:** 

Boys (n=1423)							
Age	n	BW [kg]		H [cm]		BSA [m^2^]	
		X̅ ± SD	Median (95% CI)	X̅ ± SD	Median (95% CI)	X̅ ± SD	Median (95% CI)
6	42	25.11 ± 6.33	23.4 (21.9 - 24.7)	121.64 ± 5.75	121 (117.56 - 121.5)	0.91 ± 0.12	0.89 (0.86 - 0.92)
7	261	26.39 ± 5.13	25.5 (25 - 26.1)	126.83 ± 5.69	127.5 (126 - 128)	0.96 ± 0.1	0.96 (0.93 - 0.97)
8	285	29.81 ± 6.79	28 (27 - 28)	131.65 ± 6.33	131 (130 - 131)	1.04 ± 0.13	1.02 (1 - 1.03)
9	243	34.16 ± 8.39	32 (30.2 - 32)	137.5 ± 6.13	137 (136 - 137)	1.14 ± 0.14	1.11 (1.09 - 1.12)
10	230	38.73 ± 8.45	37.65 (36 - 39)	143.18 ± 6.58	144 (141 - 144)	1.24 ± 0.14	1.23 (1.21 - 1.25)
11	179	42.34 ± 10.35	39 (37 - 39)	147.85 ± 7.62	148 (145 - 149)	1.32 ± 0.17	1.28 (1.25 - 1.31)
12	153	45.57 ± 9.7	44 (41 - 44)	153.12 ± 6.95	152 (150 - 151)	1.39 ± 0.16	1.37 (1.34 - 1.41)
13	30	50.5 ± 9.97	49.5 (45 - 54)	158.77 ± 6.65	158 (152 - 158)	1.49 ± 0.15	1.52 (1.41 - 1.56)

n, number of participants; BW, body weight; H, body height; BSA, body surface area; X̅ ± SD, arithmetic mean ± standard deviation; Median (95% CI), median value with 95% confidence interval; CI, confidence interval.

**Table 3** presents the age-specific UIC values, showing minor age-related variation, with median UIC concentrations consistently above 100 µg/L across all age groups.

**Table 3:** Age-specific urinary iodine concentration (UIC) values in Polish school-aged children.

Age	n	UIC			
		X̅	SD	Median (P50)	95% CI median
6	97	128	73	122	102.97 - 133.04
7	524	133	80.8	119	112 - 126.3
8	580	134	82.6	120	111.07 - 124.26
9	467	127	80.8	106	97.93 - 116.59
10	430	131	74.4	119	111.93 - 127.33
11	345	122	65.9	108	100.77 - 114.62
12	327	113	61.4	103	96.2 - 108.25
13	73	121	52.7	116	92 - 127

n, number of participants; BW, body weight; H, body height; BSA, body surface area; X̅, arithmetic mean; SD, standard deviation; Median (95% CI), median value with 95% confidence interval; CI, confidence interval.

**Figure 1:**
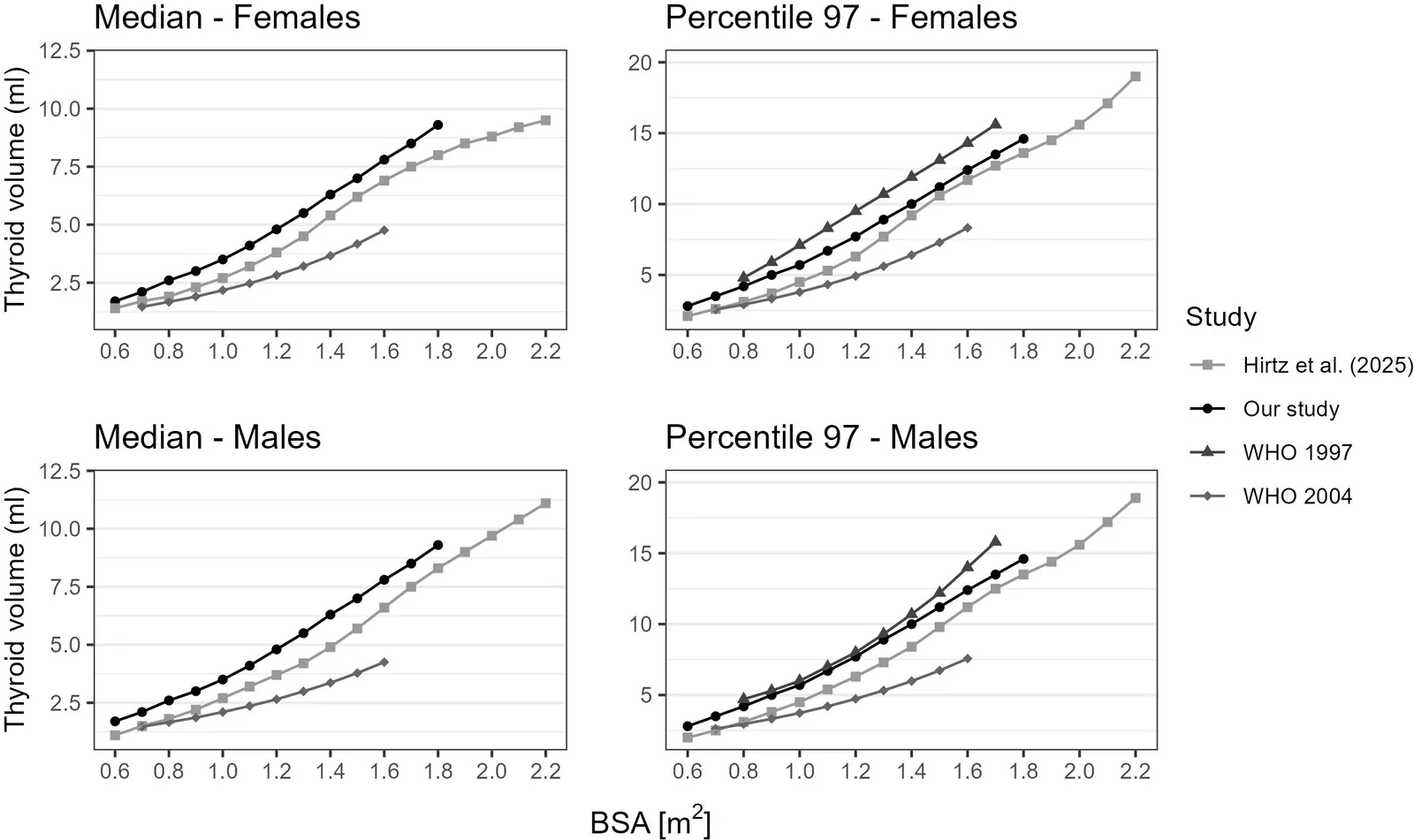
Thyroid volume by body surface area (BSA) in Polish children compared with WHO and German reference data (5, 16, 17).

**Figure 2:**
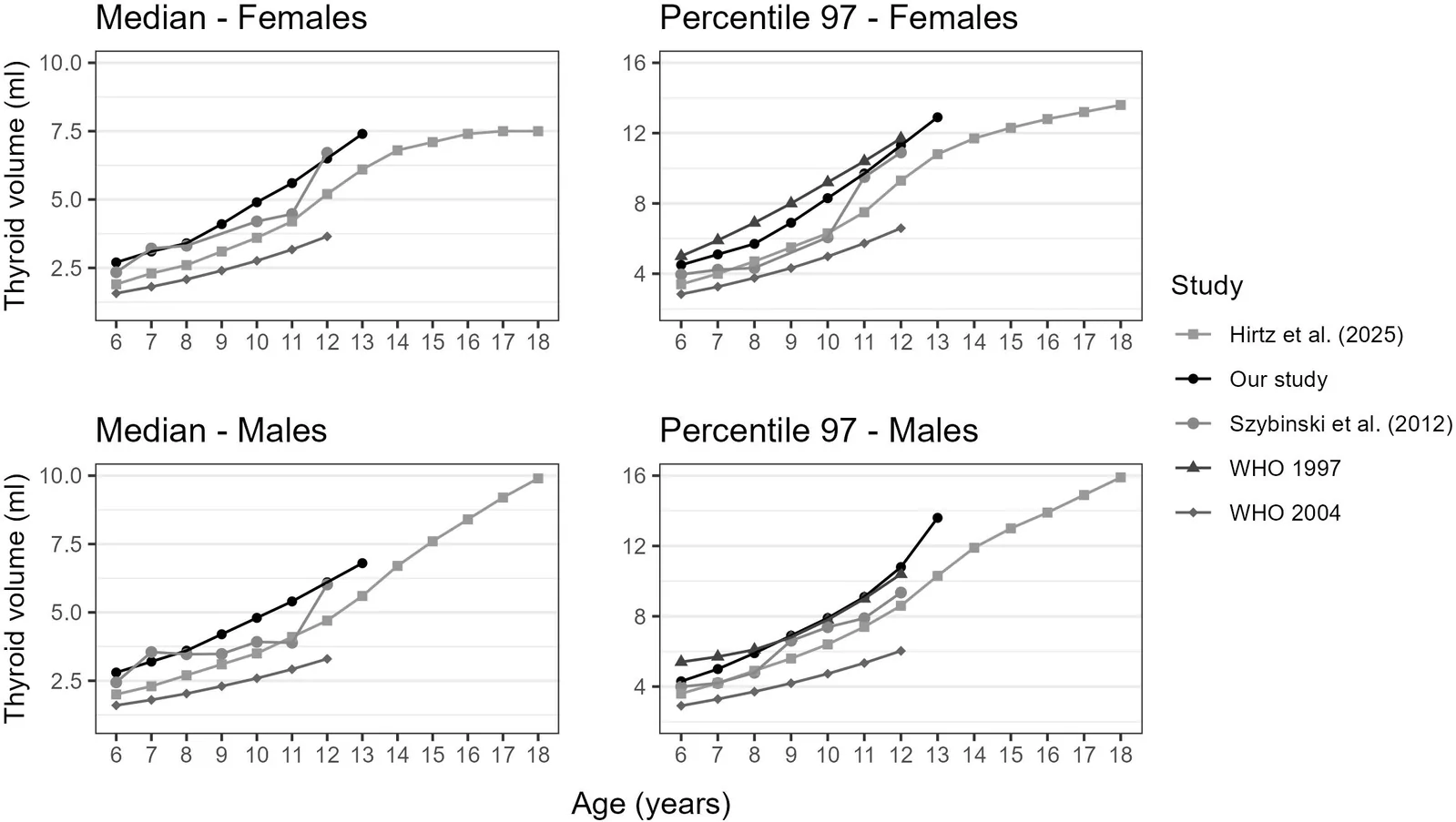
Thyroid volume by age in Polish children compared with WHO, Polish reference (2012), and German reference (2025) data (5, 8, 16, 17).

**Figures 1**, **2** present a comparison between the Tvol reference values for BSA and age, respectively, derived from our Polish cohort and those reported in previous Polish studies (8), the WHO reference charts (1997, 2004), and other contemporary European populations (5, 16, 17). The local reference values proposed in our study are lower than the WHO/International Council for the Control of Iodine Deficiency Disorders (ICCIDD) 1997 standards (16), comparable to those reported for the German population (17), and higher than the WHO/ICCIDD 2004 reference charts (5).

Age- and BSA–specific reference percentiles (P3–P97) for Tvol are presented in **Table 4**.

**Table 4:** Age- and body surface area–specific reference percentiles (P3–P97) for thyroid volume (Tvol) in Polish children by sex.

Age	P3	P10	P25	P50	P75	P90	P97
Females							
6	1.5	1.9	2.2	2.7	3.3	3.9	4.5
7	1.7	2.1	2,5	3.1	3.7	4.4	5.1
8	1.8	2.3	2.8	3.4	4.2	4.9	5.7
9	2.1	2.7	3.3	4.1	5.0	5.9	6.9
10	2.5	3.2	3.9	4.9	6.0	7.1	8.3
11	2.8	3.6	4.5	5.6	6.9	8.2	9.7
12	3.2	4.1	5.2	6.5	8.0	9.6	11.3
13	3.5	4.6	5.8	7.4	9.1	10.9	12.9
Males							
6	1.3	1.8	2.3	2.8	3.4	3.8	4.3
7	1.7	2.1	2.6	3.2	3.8	4.4	5.0
8	2.0	2.4	3.0	3.6	4.4	5.1	5.9
9	2.3	2.8	3.4	4.2	5.1	6.0	6.9
10	2.5	3.2	3.9	4.8	5.9	6.8	7.9
11	2.9	3.6	4.4	5.4	6.6	7.8	9.1
12	3.5	4.1	5.0	6.1	7.5	9.0	10.8
13	4.1	4.7	5.6	6.8	8.4	10.6	13.6
BSA	P3	P10	P25	P50	P75	P90	P97
Males							
0.6	1.1	1.4	1.6	2.0	2.4	2.8	3.2
0.8	1.5	1.8	2.2	2.7	3.2	3.8	4.3
1	2.0	2.4	2.9	3.5	4.2	4.9	5.7
1.2	2.6	3.2	3.8	4.6	5.6	6.5	7.5
1.4	3.2	3.9	4.7	5.8	6.9	8.1	9.4
1.6	3.9	4.7	5.7	6.9	8.3	9.7	11.3
1.8	4.5	5.6	6.7	8.2	9.8	11.5	13.3
Females							
0.6	0.9	1.1	1.4	1.7	2.0	2.4	2.8
0.8	1.4	1.8	2.1	2.6	3.1	3.7	4.2
1	1.9	2.4	2.9	3.5	4.3	5.0	5.7
1.2	2.6	3.2	3.9	4.8	5.8	6.7	7.7
1.4	3.4	4.2	5.1	6.3	7.5	8.8	10.0
1.6	4.1	5.2	6.4	7.8	9.3	10.8	12.4
1.8	4.8	6.1	7.5	9.3	11.1	12.8	14.6

Observed Tvol values expressed as mean ± SD by age and BSA are provided in **Supplementary Table 4**.

The distribution of the BSA-complete cohort across BSA strata, including sex-specific counts and empirical Tvol percentiles within each BSA interval, is provided in **Supplementary Table 3**.

The age- and sex-specific P97 values were considered the upper limit of normal Tvol. Among girls, the median Tvol (P50) increased gradually from 2.7 mL at age 6 to 7.4 mL at age 13, while the corresponding P97 rose from 4.5 mL to 12.9 mL over the same period.

**Figure 3:**
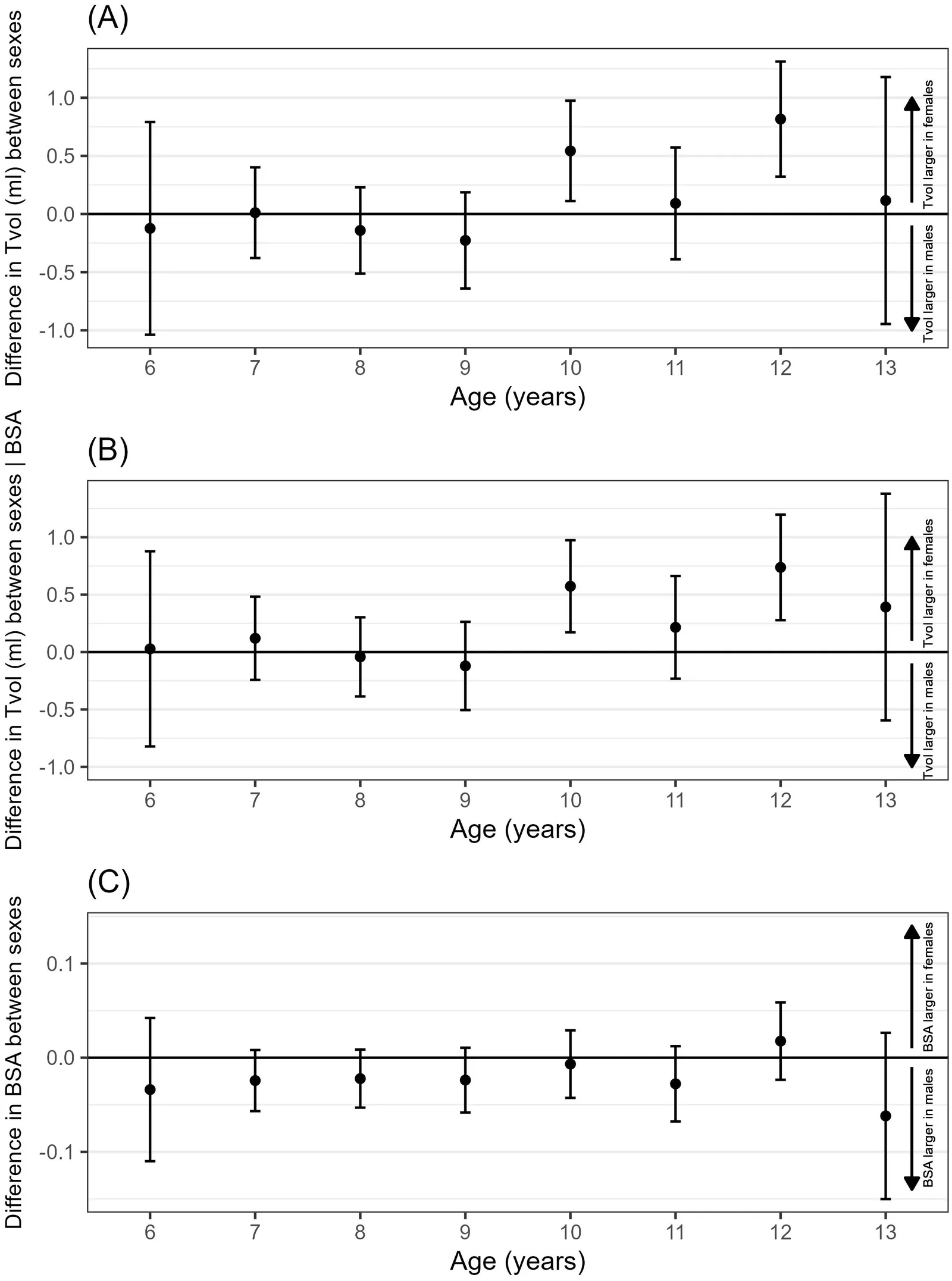
Sex-related differences in thyroid volume (Tvol) and body surface area (BSA) across age groups. **(A)** Difference between sexes for total thyroid volume in relation to age. **(B)** Difference between sexes for total thyroid volume in relation to age after adjustment for BSA by analysis of covariance. **(C)** Difference in BSA between sexes in relation to age. Points indicate estimated differences and error bars represent 95% confidence intervals. Confidence intervals were adjusted for multiple comparisons using Holm’s method.

Sex-related differences in Tvol and BSA across age groups are presented in **Figures 3A–C**.

The results of the multiple linear regression are presented in **Table 5**. The final model explained 50.24% of Tvol variance. BSA emerged as the strongest independent determinant of Tvol (β = 0.46, p < 0.0001), followed by age (β = 0.29, p < 0.0001). Male sex showed a minor negative association (β = -0.05, p = 0.0004), indicating slightly smaller thyroid volumes in boys after adjustment for BSA and age. An alternative model using BMI instead of BSA explained less variance (R^2^ = 46.23%; **Supplementary Table 2**), supporting BSA as the main anthropometric scaling variable.

**Table 5:** Results of multivariable linear regression analysis for predictors of Tvol in Polish school-aged children.

Predictor	B	β	rsemi	t(2835)	% of variance	p
Intercept	-3.07			-20.63		<0.0001
Sex = male	-0.18	-0.05	-0.07	-3.57	0.44	0.0004
Age	0.31	0.29	0.09	13.68	2.46	<0.0001
BSA	4.26	0.46	0.10	21.98	5.78	<0.0001

B - unstandardized regression coefficient; β - standardized regression coefficient; rsemi - semi-partial correlation coefficient; t(2835) – t statistic with 2,835 degrees of freedom; % of variance - proportion of explained variance; p - level of statistical significance; BSA - body surface area. The model’s R^2^ was 50.24%.

## Discussion

Epidemiological surveys in 1992–1993 revealed moderate iodine deficiency across almost the entire territory of Poland (18). Following the recommendations of the Polish Council for Control of Iodine Deficiency Disorders (PCCIDD), in 1997 Poland introduced mandatory iodization of household salt (20–40 mg KI/kg) (19). This model proved effective: the prevalence of goiter in school-aged children fell below endemic levels, and since the late 1990s Poland has been considered an iodine-sufficient country (20, 21). Zygmunt et al. compared schoolchildren examined in 1994, 1999, 2010, and 2016 in one town in central Poland and observed a significant rise in median UIC to values indicating iodine sufficiency; goiter prevalence, expressed as the TV-to-BSA ratio above the WHO reference, fell from 92.6% in 1994 to 18.5% in 1999, 15.8% in 2010, and 11.6% in 2016 (20). According to the previous Polish national reference, goiter prevalence by age and sex decreased from 66.6% in 1994 to 2.5% in 2016 (8).

According to WHO epidemiological criteria for school-aged children, population median UIC values <20 µg/L indicate severe iodine deficiency, 20-49 µg/L moderate deficiency, 50-99 µg/L mild deficiency, 100-199 µg/L adequate iodine nutrition, 200-299 µg/L iodine intake above requirements, and ≥300 µg/L excessive iodine intake (24). In the present study, iodine sufficiency was defined at the population level using median UIC ≥100 µg/L in the corresponding school or region.

Concerning Tvol reference values for Polish children and adolescents, there has been a notable absence of representative studies for almost 15 years. In 2012, Szybiński et al. established Tvol reference values for Polish school-aged children, which have since been used as the national standard in pediatric examinations (8). The mentioned study was conducted between 2006 and 2007 in coastal regions of Poland with the highest iodine supply and included only children (n = 642; ages 6-12) with UIC in a casual morning urine sample ≥100 µg/L. The reference volumes reported were lower than those established in 1997 by the WHO (16), but higher than the international WHO values from 2004 (5).

Zimmermann et al. emphasized that Tvol reference values should be established in children living in regions of sustained iodine sufficiency (5). In a study by Aghini-Lombardi et al., median Tvol was significantly higher in children born before the introduction of iodine prophylaxis than in those born after it (22). In the work by Szybiński et al., only children aged 6–8 years met the criterion of being born after 1997 (8). The authors selected children living in coastal regions of Poland because some seaside towns had already been reported as iodine sufficient in 1994/1995 (23). Moreover, when selecting the study population, the authors used an inclusion criterion of UIC ≥100 µg/L from a single spot urine sample, rather than considering the median UIC of the entire population (5, 24). Classifying every individual with a spot UIC below 100 µg/L as iodine-deficient is a frequent misinterpretation. Because daily iodine intake and UIC vary considerably, even individuals with adequate iodine consumption may occasionally present with low UIC values. Therefore, in iodine-sufficient populations, some samples will fall below 100 µg/L, and such isolated results should not be interpreted as iodine deficiency. Likewise, a single UIC above 100 µg/L does not confirm individual sufficiency (25). To reduce misclassification, we included only regions with median UIC ≥100 µg/L and children born after nationwide iodine prophylaxis was introduced in 1997.

Although our study population was born after the implementation of mandatory iodine prophylaxis in 1997, Tvol values in our cohort were consistently higher across all age groups and both sexes than those reported by Szybiński et al. (8). This may be due to (a) preselection bias in Szybiński’s manuscript, (b) a longer history of iodine sufficiency in the seaside area, or (c) a secular trend in the body composition of schoolchildren. In a large cross-sectional study of 13,172 boys from Eastern Poland (1986-2021), Wasiluk and Saczuk reported significant increases in height, body mass, and BMI across all age groups (9). Kułaga et al., in a nationwide cross-sectional study of over 17,000 Polish schoolchildren (the OLAF project), demonstrated a significant secular increase in height, body weight, and BMI compared with earlier national data (26). This upward trend in body size likely explains the larger age-adjusted thyroid volume seen in modern pediatric populations compared with earlier datasets.

Since the previous reference charts for thyroid volume in Polish school-aged children did not include values normalized to BSA, we aimed to establish reference values for Tvol relative to BSA in our cohort.

BSA is considered a key anthropometric predictor of thyroid size in children and adults (1, 3, 27). In the study by Folić et al., which included 535 healthy children aged 4–18 years from iodine-sufficient regions of Serbia, Tvol showed significant positive correlations with age (r = 0.64), height (r = 0.73), weight (r = 0.74), BMI (r = 0.55), and most strongly with body surface area (BSA; r = 0.77), with age remaining an independent positive predictor of thyroid volume in multiple regression analysis (1). Kaloumenou et al. stated in a study group of 440 schoolchildren aged 5–18 years, that BSA correlated with Tvol in boys (r = 0.730, p < 0.0005) and girls (r = 0.623, p < 0.0005) (27).

Consistent with these findings, multiple regression analysis in our study identified BSA as the strongest predictor of thyroid volume (β = 0.46, p < 0.0001), and the BSA-based model explained more variance than the alternative BMI-based model (R^2^ = 50.24% vs. 46.23%). The detailed BMI-based model is shown in **Supplementary Table 2**.

In their systematic review, Johnson et al. analyzed 27 studies in iodine-sufficient school-aged populations and emphasized BSA as a key determinant of thyroid volume variability (3). Most contemporary pediatric reference datasets incorporate BSA—or combinations of height and weight—to reduce interindividual variance and improve comparability. The WHO/ICCIDD (2004) reference standards were also normalized to BSA, underscoring the importance of relating Tvol to body size when establishing or interpreting reference values.

In our cohort, thyroid volume showed a gradual age-related increase in both sexes.

After adjustment for BSA and age, boys showed marginally lower Tvol than girls (β = -0.05, p = 0.0004), but the effect size was small. Our results are consistent with recent European data indicating that apparent sex differences in Tvol largely reflect differences in body size rather than intrinsic biological variation. In the WHO guidelines, after normalization for BSA, differences between thyroid volume in boys and girls were minimal (5). Hirtz et al. generated reference charts for German children and adolescents and demonstrated that girls showed transiently higher Tvol at the onset of puberty (around ages 11-13), whereas boys exceeded girls later (≥14 years), reflecting differences in pubertal timing and hormonal dynamics rather than true sexual dimorphism (17). Comparable findings were reported in Spanish and Dutch populations, in which girls in the 11-12-year-old group showed larger Tvol (28, 29).

Fleury et al. interpreted sex differences in Tvol values as being related to differences in pubertal timing (30). Earlier puberty onset in girls means girls enter the hormonal environment (higher estrogen, IGF-1, growth hormone surge) sooner than boys. That exposure could trigger earlier thyroid follicular growth. Estrogen can stimulate the proliferation of thyrocytes via estrogen receptors, thus possibly contributing to accelerated thyroid development in girls during puberty (31).

The WHO/ICCIDD (2004) reference charts for Tvol may underestimate normal thyroid size in children, as they were derived from studies conducted in the 1990s using different methodologies and study populations. The cohort was highly heterogeneous, including children from regions (with Swiss children as the European representative) with variable iodine intake and wide fluctuations in UIC. Although the median UIC values in all regions indicated iodine sufficiency, substantial interregional differences were observed, with both low (<50 µg/L) and excessively high (>2000 µg/L) values reported. Such variability likely influenced thyroid size, therefore the authors have emphasized that population-specific reference ranges are preferable, particularly in countries with longstanding iodine sufficiency, where local anthropometric and environmental factors might influence Tvol the most (5).

As noted by Hirtz et al., the WHO 2004 reference charts provide estimates of TV approximately 20-30% lower than values reported in more recent studies from European countries such as Germany, Sweden, and Spain (17, 28, 32).

Our reference values are comparable to those reported in iodine-sufficient German children analyzed by Hirtz et al. (17). However, despite earlier improvement from mild-to-moderate deficiency, recent data indicate declining iodine status in Germany, with median daily excretion decreasing from ~84.8 µg/day (2003–2012) to ~58.9 µg/day by 2018 (33).

Our proposed reference values closely match those reported for Swedish children and adolescents aged 6 to 13 years. Sweden has maintained nationwide salt iodization since 1936, with documented iodine sufficiency already in the late 1980s (32, 34). Therefore, the comparable Tvol values observed in our cohort may support the statement that Poland has now achieved stable iodine sufficiency.

Considering that our BSA-adjusted percentile charts for Tvol in children aged 6–13 years were developed from a large iodine-sufficient cohort and are consistent with data from neighboring countries, they may serve as population-specific charts for everyday pediatric ultrasonography.

## Limitations

We did not quantify inter-observer and intra-observer variability in Tvol measurements for this study. Previous investigations have demonstrated that sonographic measurement of Tvol in children is subject to considerable variability (mean intra-observer variation approximately 8% ± 6.7% and inter-observer variation approximately 13.3% ± 8.2%), which may introduce measurement error (35). Zygmunt et al. demonstrated that excessive inter-observer variability in the absence of strictly standardized measurement protocols can lead to erroneous interpretation of ultrasound results; in their study, inter-observer variability for total Tvol reached 34-36%, and intra-observer variability reached 9-10% (36). In addition, BSA was unavailable for 327 children in the final Tvol cohort; however, the BSA-complete subset retained 89.7% of children and was used only for analyses requiring anthropometry. The number of children was lower in the highest BSA strata; therefore, BSA-specific percentile estimates close to the upper BSA boundary should be interpreted with appropriate caution (**Supplementary Table 3**).

Methodological aspects of ultrasound examination may introduce systematic measurement bias. Zygmunt et al. showed that Tvol measured in the sitting position was 0.4–0.6 mL lower than in the supine position (12–15% difference), demonstrating a clinically relevant effect of body position on thyroid size estimates (36). As the reference values by Zimmermann et al. were derived from examinations performed in the sitting position (5), applying them to supine measurements may lead to misclassification and overestimation of goiter prevalence.

Although Poland has been classified as iodine-sufficient since the late 1990s, recent nutritional and public health data suggest a declining trend in salt consumption, potentially affecting iodine intake at the population level. Poland has historically ranked among the highest salt-consuming countries in the European Union (average approximately 11.5 g/day) (37). Global initiatives aim for a 30% reduction in salt intake (38). Thus, the long-term impact of decreasing dietary salt and iodine intake cannot be fully excluded. In a recent nationwide study, school-aged children remained iodine-sufficient (median UIC 119.8 µg/L), whereas vulnerable groups of pregnant women (median UIC 111.6 µg/L; WHO adequacy range 150-249 µg/L) and lactating women (median UIC 68.0 µg/L; WHO adequacy range ≥100 µg/L) showed insufficient iodine intake (39).

Finally, the present percentile charts apply to school-aged children between 6 and 13 years of age. They should not be extrapolated to younger children, and future studies including children under 6 years of age would be clinically valuable for pediatric endocrinology and ultrasound practice.

## Conclusion

This nationwide study provides age- and BSA-specific percentile charts for Tvol in Polish school-aged children born after the implementation of universal iodine prophylaxis. These charts, derived from a large iodine-sufficient cohort, align closely with recent European data from Germany and Sweden and complement older national and WHO reference values. They provide clinically relevant population-specific standards for pediatric thyroid ultrasonography in Poland and may be useful for interpreting ultrasound findings in other Central European populations with similar nutritional profiles.

## Data Availability

The data analyzed in this study is subject to the following licenses/restrictions: The datasets analyzed in this study are not publicly available because they contain de-identified but sensitive health-related data from children collected within the Polish National Program for the Elimination of Iodine Deficiency. Data sharing is subject to institutional, ethical, and data-protection regulations. Requests to access the datasets should be directed to the corresponding author and will be considered on a reasonable request basis, subject to approval by the responsible institution and a data-sharing agreement. Requests to access these datasets should be directed to Małgorzata Trofimiuk-Muldner, malgorzata.trofimiuk@uj.edu.pl.
