# Supplementary material for: Age- and body surface area-specific ultrasound percentile charts for thyroid volume in Polish school-aged children: comparison with European cohorts
Source: Front Endocrinol (Lausanne). 2026 Sep 7;17:1945848. doi: 10.3389/fendo.2026.1945848 (PMC13590292; doi:10.3389/fendo.2026.1945848)
Supplement: Supplementary file 1 [file SupplementaryFile1.docx]

**Supplementary Material**

The following supplementary material provides additional descriptive and modelling information supporting the main manuscript.

**Supplementary Table S1. Characteristics of excluded and included participants.**

| **Variable** | **Total excluded dataset, n = 7,286** | **Excluded due to date of birth before 1998, n = 5,615** | **Excluded due to region median UIC <100 µg/L, n = 1,389** | **Final dataset included n = 3,170** |
| --- | --- | --- | --- | --- |
| **Tvol, mL** | 4.56 (3.36-6.20) | 4.81 (3.48-6.61) | 3.93 (3.04-4.81) | 4.20 (3.18-5.59) |
| **Tvol/BSA ratio, mL/m²** | 4.01 (3.11-4.98) | 4.10 (3.17-5.14) | 3.74 (3.01-4.50) | 3.96 (3.16-5.07) |
| **Age, years** | 9.81 (8.15-11.60) | 10.31 (8.43-11.98) | 8.17 (7.32-9.37) | 9.04 (7.67-10.63) |
| **BSA, m²** | 1.15 (1.00-1.32) | 1.18 (1.03-1.35) | 1.04 (0.94-1.17) | 1.08 (0.93-1.26) |
| **UIC, µg/L** | 91.0 (60.71-135.40) | 91.0 (60.14-135.50) | 89.4 (60.44-130.62) | 113.4 (76.49-162.89) |
| **BMI, kg/m²** | 17.10 (15.57-19.29) | 17.16 (15.61-19.35) | 16.71 (15.38-18.63) | 17.05 (15.38-19.31) |
| **Boys, %** | 48.1 | 48.0 | 49.7 | 49.4 |

**Note.** Values are presented as median (1^st^ quartile – 3^rd^ quartile) unless otherwise indicated. The finally included cohort was younger, had smaller unstandardized thyroid volumes, and had higher UIC than the overall excluded group, which reflects the predefined selection of children born after implementation of mandatory iodine prophylaxis and from iodine-sufficient regions. The Tvol/BSA ratio was similar between the total excluded dataset and the final analytical cohort.

**Supplementary Table S2. Alternative multivariable regression model replacing BSA with BMI.**

|  | **B** | **β** | **rsemi** | **t** | **% of variance** | **p** |
| --- | --- | --- | --- | --- | --- | --- |
| **Intercept** | -3.46 |  |  | -19.35 |  | <0.0001 |
| **Sex = male** | -0.16 | -0.04 | -0.07 | -2.99 | 0.31 | 0.0029 |
| **Age** | 0.62 | 0.57 | 0.09 | 38.58 | 30.24 | <0.0001 |
| **BMI** | 0.14 | 0.23 | 0.10 | 15.35 | 6.71 | <0.0001 |

**Note.** The BMI-based model explained 46.23% of Tvol variance. In the main BSA-based model presented in the manuscript, the corresponding R² was 50.24%, supporting BSA as the preferred anthropometric scaling variable. B, unstandardized regression coefficient; β, standardized regression coefficient; rsemi, semi-partial correlation coefficient; BMI, body mass index.

**Supplementary Table S3. Distribution of children across body surface area (BSA) strata in the BSA-complete cohort.**

| **BSA stratum (m²)** | **Girls, n** | **Boys, n** | **Total, n** | **% of BSA cohort** | **Median age, years** | **Tvol P50, mL** | **Tvol P3, mL** | **Tvol P97, mL** |
| --- | --- | --- | --- | --- | --- | --- | --- | --- |
| (0.6, 0.8] | 25 | 13 | 38 | 1.3 | 6.95 | 2.47 | 1.83 | 3.37 |
| (0.8, 1.0] | 410 | 368 | 778 | 27.4 | 7.43 | 3.15 | 1.68 | 5.32 |
| (1.0, 1.2] | 482 | 488 | 970 | 34.1 | 8.75 | 3.98 | 2.15 | 7.16 |
| (1.2, 1.4] | 316 | 374 | 690 | 24.3 | 10.42 | 5.38 | 2.88 | 9.22 |
| (1.4, 1.6] | 150 | 134 | 284 | 10.0 | 11.61 | 6.20 | 3.59 | 11.74 |
| (1.6, 1.8] | 37 | 46 | 83 | 2.9 | 11.88 | 7.96 | 4.71 | 15.13 |
| **Total** | **1420** | **1423** | **2843** | **100.0** |  |  |  |  |

Note. Values are based on the BSA-complete cohort (n = 2,843; 1,420 girls and 1,423 boys). Percentages were calculated relative to the BSA-complete cohort. The distribution shows fewer observations in the highest BSA intervals, supporting cautious interpretation of upper-range BSA-specific percentile estimates. Tvol, total thyroid volume; P3/P50/P97, 3rd/50th/97th percentile.

**Supplementary Table S4. Thyroid volume by age and body surface area expressed as mean ( SD).**

| **Group** | **Mean (SD) – Boys** | **Mean (SD) – Girls** | **Winsorized mean (SD) – Boys** | **Winsorized mean (SD) – Girls** |
| --- | --- | --- | --- | --- |
| **Age, years** |  |  |  |  |
| 6 | 3.15 (0.89) | 3.19 (1.76) | 3.13 (0.85) | 3.08 (0.84) |
| 7 | 3.51 (1.14) | 3.29 (1.07) | 3.47 (0.99) | 3.27 (0.98) |
| 8 | 3.94 (1.18) | 3.88 (1.19) | 3.93 (1.15) | 3.87 (1.16) |
| 9 | 4.72 (1.52) | 4.68 (1.79) | 4.70 (1.46) | 4.60 (1.40) |
| 10 | 5.19 (1.46) | 5.53 (2.06) | 5.18 (1.41) | 5.43 (1.77) |
| 11 | 5.88 (1.74) | 6.22 (2.64) | 5.87 (1.72) | 6.05 (1.84) |
| 12 | 7.08 (3.09) | 7.40 (2.64) | 6.91 (2.21) | 7.30 (2.32) |
| **BSA, m²** |  |  |  |  |
| 0.6 | 2.53 (0.49) | 2.47 (0.54) | 2.53 (0.49) | 2.47 (0.54) |
| 0.8 | 3.26 (0.94) | 3.27 (1.35) | 3.25 (0.91) | 3.21 (0.92) |
| 1.0 | 4.13 (1.24) | 4.25 (1.56) | 4.12 (1.21) | 4.18 (1.29) |
| 1.2 | 5.36 (1.60) | 5.65 (1.77) | 5.33 (1.52) | 5.60 (1.64) |
| 1.4 | 6.14 (1.64) | 7.32 (2.50) | 6.13 (1.63) | 7.10 (1.94) |
| 1.6 | 8.52 (3.85) | 8.43 (2.63) | 8.16 (2.24) | 8.27 (2.20) |

Note. Values are presented as observed mean (SD) for total thyroid volume in mL. The table includes original and winsorized descriptive values by age and BSA, separately for boys and girls, according to the statistician-generated output.

**Supplementary Figure S1**. Prototype interface of the thyroid-volume prediction tool based on the multivariable percentile model.

Comment. The prototype application visualizes a child's measured thyroid volume against the cohort-derived predicted distribution using age, sex, height, weight, and measured Tvol as user inputs. The red vertical line indicates the child's measured thyroid volume, and the table below the plot shows the predicted P3, P10, P25, P50, P75, P90, and P97 values. The tool is intended only as a supportive visualization of the statistical model and should not be used as a standalone diagnostic test.
